# Occurrence and subtyping of the intestinal protozoan *Blastocystis* sp. in French oyster areas reveal potential fecal contamination routes and associated risks to consumer health

**DOI:** 10.1016/j.fawpar.2026.e00332

**Published:** 2026-03-19

**Authors:** Manon Ryckman, Constance Denoyelle, Cyrielle Lecadet, Nausicaa Gantois, Jeremy Desramaut, Ruben Garcia Dominguez, Magali Chabé, Luen-Luen Li, Sébastien Monchy, Gabriela Certad, Isabelle Arzul, Eric Viscogliosi

**Affiliations:** a Univ. Lille, CNRS, Inserm, CHU Lille, Institut Pasteur de Lille, U1019 – UMR 9017 – CIIL – Centre d'Infection et d'Immunité de Lille, Lille, France; b Université du Littoral Côte d'Opale, CNRS, Univ. Lille, UMR 8187, LOG, Laboratoire d'Océanologie et de Géosciences, Wimereux, France; c Adaptation et Santé des Invertébrés Marins (ASIM), Ifremer, La Tremblade, France; d Centro Andaluz de Biología del Desarrollo, CSIC, Universidad Pablo de Olavide, Sevilla, Spain; e Délégation à la Recherche Clinique et à l'Innovation, Groupement des Hôpitaux de l'Institut Catholique de Lille, Lille, France

**Keywords:** *Blastocystis* sp., Intestinal protozoa, Oysters, Molecular epidemiology, Fecal pollution, Public health risk

## Abstract

*Blastocystis* sp. is the most frequently detected intestinal protozoan in humans and recognized as a zoonotic eukaryote. Therefore, raw-consumed mollusks such as oysters may act as potential reservoirs for contamination, posing a risk to consumer health. However, data on the prevalence and subtype (ST) distribution of this parasite in mollusks remain scarce. This study aimed to detect *Blastocystis* sp. by real-time PCR in 360 wild oysters collected in 2021 from three major French oyster areas (Arcachon, Bourgneuf, and Marennes-Oléron bays) and in 210 surrounding water samples (nano- and micro-mesoplankton fractions). Additionally, 120 oysters collected in 2024 from Marennes-Oléron were screened to complement the analysis. The prevalence observed in oysters was 1.4% in 2021 and increased to 19.2% in 2024, while only 1.9% of environmental samples tested positive. Sanger and next-generation sequencing of PCR products from positive samples identified ten STs in oysters (ST1, ST2, ST3, ST4, ST6, ST7, ST21, ST26, ST30, ST44), four in nanoplankton (ST4, ST6, ST7, ST28), and one in micro-mesoplankton (ST7), indicating circulation of the parasite in the marine environment while highlighting the role of oysters as sentinels of microbiological quality in coastal ecosystems. The ST diversity and absence of a predominant ST reported in oysters suggest that these bivalves are not natural hosts but rather passive carriers of *Blastocystis* sp. The ST profiles suggest fecal contamination originating from both humans and animals (bovines and birds). Since most STs identified are transmissible to humans, the consumption of raw contaminated oysters may represent a potential public health risk.

## Highlights

• *Blastocystis* sp. is frequently detected in French oyster production areas.
• *Blastocystis* sp. STs analysis suggests oysters are not natural hosts of the parasite.
• Contamination of oysters is related to fecal pollution from human and animal sources.
• Oysters serve as reliable sentinels of the microbiological quality of their ecosystem.
• Consumption of raw contaminated oysters may pose a risk to human health.

## 1 Introduction

*Blastocystis* sp. is an anaerobic intestinal protozoan belonging to the highly heterogeneous group of stramenopiles (Jirsova and Wideman, 2024). All recent epidemiological surveys using molecular tools demonstrate that *Blastocystis* sp. is by far the most frequently found unicellular eukaryote in human stool, with between one and two billion people colonized worldwide (Scanlan and Stensvold, 2013; Andersen and Stensvold, 2016). It has also been identified in a wide range of animal groups, from insects to mammals, often with significant prevalence rates (Hublin et al., 2021; Rauff-Adetotun et al., 2020, Rauff-Adetotun et al., 2021; Sangarri et al., 2022). Transmitted through fecal contamination, primarily via the consumption of water but also food contaminated with the environmental resistant cystic forms of the protozoan (Tan, 2008; Didban et al., 2025), its prevalence can thus reach an average of around 20% in European countries (Moniot et al., 2024) and largely exceed 50% in populations of developing countries combining precarious sanitary conditions and ineffective water treatment, particularly in Africa (Khaled et al., 2020; Guilavogui et al., 2022).

The genus *Blastocystis* is characterized by a broad genetic diversity based on comparisons of small subunit (SSU) rDNA gene sequences, with the current identification of 45 lineages called subtypes (STs) in birds and mammals alone (Figueiredo et al., 2025). Among these STs, four (ST1-ST4) are most commonly found in the human population, primarily circulating through widespread human-to-human transmission (Alfellani et al., 2013a; Jimenez et al., 2019; Rauff-Adetotun et al., 2021; Fusaro et al., 2024). Other STs, however, are considered to have an animal origin due to their high frequency in certain animal groups. For example, ST6 and ST7 are frequently found in birds, ST10 and ST14 in cattle and small ruminants, and ST5 in pigs (Jimenez et al., 2019; Hublin et al., 2021; Barati et al., 2022; Shams et al., 2021; Shams et al., 2022; Rehena et al., 2025). Interestingly, a significant proportion of human isolates belong to these animal-derived STs, indicating zoonotic transmission (Stensvold and Clark, 2016). Accordingly, numerous surveys have confirmed this mode of transmission with the sharing of identical *Blastocystis* sp. isolates, for example, between livestock and their farmers (Greige et al., 2018), zoo-housed non-human primates and their caregivers (Alfellani et al., 2013b; Šejnohová et al., 2024) or domestic pigs and piggery workers (Wang et al., 2014).

As most individuals colonized by *Blastocystis* sp. remain asymptomatic, the pathogenic potential of this protozoan has long been a subject of debate. However, reports indicate that in some cases where the parasite was the only potential pathogen identified in patients with diarrhea, the condition resolved following anti-parasitic treatment (Fréalle et al., 2015). Moreover, evidence supporting the potential pathogenicity of *Blastocystis* sp. has also emerged from both in vitro and in vivo studies, and appears to be ST-dependent (Ajjampur and Tan, 2016; Deng et al., 2021; Deng and Tan, 2025). Briefly, the zoonotic avian ST7 can compromise gut barrier integrity by disrupting tight junctions, resulting in increased epithelial permeability — a feature not observed with ST4, for example. Moreover, ST7 has been shown to promote gut dysbiosis by reducing beneficial bacterial populations and triggering pro-inflammatory responses. In contrast, colonization by ST1, ST3 and ST4 is associated with increased gut microbiota diversity and the promotion of an anti-inflammatory environment in the intestinal mucosa (Billy et al., 2021; Deng et al., 2022; Aykur et al., 2024; Antonetti et al., 2024; Huang et al., 2024; Deng and Tan, 2025).

Zoonotic transmission requires close and frequent contact between humans and animal hosts colonized by *Blastocystis* sp. Therefore, most epidemiological surveys have naturally focused on livestock, particularly herbivores and poultry, to identify potential sources of contamination for humans (Greige et al., 2018; Naguib et al., 2022; Figueiredo et al., 2024; Naguib et al., 2024; Figueiredo et al., 2025). However, other animal groups may also represent a significant risk due to their handling and/or consumption. This is the case for marine animals such as fish and mollusks, which have been the subject of only a few studies (Ghafari-Cherati et al., 2024), even though protozoans have been identified as under-recognized foodborne pathogens transmitted through shellfish (Kim et al., 2024).

Large-scale epidemiological surveys using molecular tools have identified *Blastocystis* sp. in edible marine fish and marine mammals along the coasts of Northern France (Gantois et al., 2020), as well as in dugong populations of the Persian Gulf (Boughattas et al., 2025). Concurrently, mussels collected from the same French geographical area were found to harbor a significant burden of *Blastocystis* sp., with 62% of tested individuals colonized (Ryckman et al., 2024). However, as these mollusks are more often consumed cooked the risk of transmission to humans appeared to be limited. In contrast, other seafood products such as oysters are generally consumed raw, thus increasing the potential risk of direct transmission for consumers. The risk is even greater since bivalve mollusks have the ability to filter large quantities of water daily and therefore accumulate potential contaminants such as waterborne protozoa (Giangaspero et al., 2019). This issue is especially pertinent in France, the leading producer and consumer of oysters in the European Union (EUMOFA, 2022), where no epidemiological data on *Blastocystis* sp. in oysters are available.

To our knowledge, only four studies, all conducted in the same geographical area around Mexico City (Martinez Barbabosa et al., 2017, Martinez Barbabosa et al., 2018, Martinez Barbabosa et al., 2021; Campos Compean et al., 2018), have reported the presence of *Blastocystis* sp. in oysters (*Crassostrea virginica*) at significant frequencies. However, only few ST1 isolates have been molecularly characterized in these mollusks from Mexico. Given the lack of molecular data regarding the prevalence and distribution of *Blastocystis* sp. STs in oysters, a survey was conducted by retrospectively screening a large collection of wild individuals collected throughout 2021 from three French oyster basins. In addition, a series of seawater oysters sampled over several months in 2024 from the French area of Marennes-Oléron was analyzed. The aim of both surveys was to evaluate the risk of zoonotic transmission of the protozoan from these bivalves for consumers. This study was complemented by the analysis of various seawater samples collected in 2021 near the oyster sites in order to clarify the circulation of *Blastocystis* sp. in these economically important French mollusk production areas.

## 2 Materials and methods

### 2.1 Oysters and environment sampling sites

**Fig. 1:**
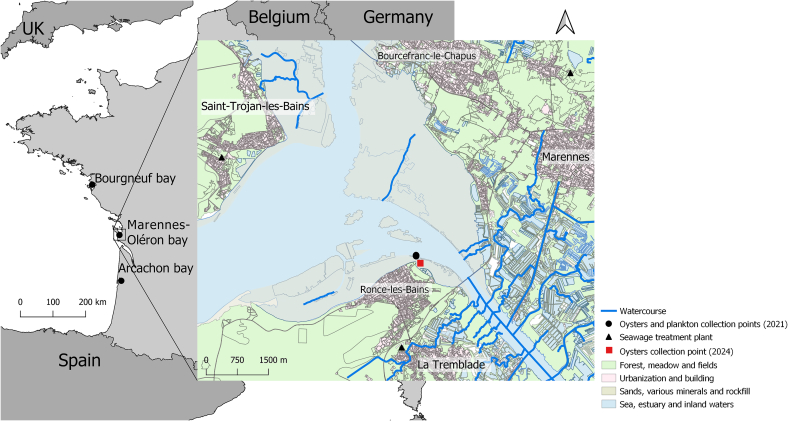
Map showing the locations of oyster and plankton sampling sites along the French Atlantic coast.

An initial oyster sampling campaign was conducted in 2021 across three basins along the French Atlantic coast, namely Gois (Bourgneuf bay, Vendée; GPS coordinates: 46.922290, −2.105864), La Floride (Marennes-Oléron bay, Charente Maritime; GPS coordinates: 45.802636, −1.151221), and Le Tès (Arcachon bay, Gironde; GPS coordinates: 44.665, −1.138299) (Fig. 1) (Arzul et al., 2026). Briefly, a total of 120 adult individuals (*Magallana gigas*) were manually and randomly collected at each site every three months over the course of one year (30 oysters per season) during low tide from wild oyster reefs. During the same period, a total of 210 seawater samples, comprising micro-mesoplankton and nanoplankton fractions, were collected below the surface from the same three sites (Arzul et al., 2026). Specifically, 80 micro-mesoplankton samples were collected using 20 μm and 200 μm nets along two 50-m transects conducted at ebb tide near the oyster reefs. Additionally, 130 nanoplankton samples were obtained from seawater collected approximately 75 m away from the oyster sites. To complete this survey, a second campaign for sampling wild oysters was conducted in 2024, with 40 oysters being collected in July, September, and November at the same Marennes-Oléron bay site as that of 2021.

### 2.2 Processing of oysters and environmental samples and DNA extraction

DNA was extracted from washed and crushed micro-mesoplankton fraction using the QIAamp DNA Mini Kit (Qiagen, Courtaboeuf, France) (Arzul et al., 2026). For nanoplankton fraction, DNA was extracted from 2.5 L of seawater prefiltered at 20 μm and then filtered through a 1 μm membrane using the DNeasy PowerWater Kit (Qiagen). For oysters sampled in 2021, DNA was extracted using the QIAamp DNA Mini Kit (Qiagen) from 20 mg of pooled sample of gills, digestive gland, and mantle tissues previously homogenized. In the case of oysters collected in 2024, individuals were transported in a freezer bag to the laboratory then stored at 4 °C until dissection. Briefly, the mollusks were opened by a scalpel section at the level of the adductor muscle, externally washed with sterile distilled water and removed from their shells before dissection on a sterile surface. The gills and the gastrointestinal tract were carefully dissected to separately collect the digestive tract and gills from each specimen. Each organ was then homogenized in an Eppendorf tube using sterile scalpels, and DNA extraction was performed from about 250 mg of dilacerated organs using the NucleoSpin 96 Soil Kit (Macherey-Nagel GmbH & Co. KG, Düren, Germany), following the manufacturer's recommended protocol.

### 2.3 PCR identification of *Blastocystis* sp. and subtyping of isolates from single infections/colonizations using Sanger sequencing

To detect *Blastocystis* sp., 2 μL of purified DNA extracted from each oyster sample, or diluted to one-tenth for environmental samples to limit PCR inhibition (Arzul et al., 2026), was analyzed by real-time PCR (qPCR). The assay employed the primer pair BL18SPPF1 (5′-AGTAGTCATACGCTCGTCTCAAA-3′) and BL18SR2PP (5′-TCTTCGTTACCCGTTACTGC-3′), which amplifies an approximately 300 bp fragment of the small subunit (SSU) rDNA gene of all known *Blastocystis* sp. STs (Poirier et al., 2011). All samples were tested in duplicate and positive (DNAs from *Blastocystis* sp. ST4 strain WR1 axenic cultures) and negative (nuclease-free water in place of DNA) controls were included in each qPCR run. A result was interpreted as negative if the cycle threshold (Ct) value was greater than 36 or if no amplification curve was observed during the reaction. Ct values were thus recorded for all qPCR-positive samples, and the corresponding amplicons were subsequently purified and sequenced using Sanger technology (Genoscreen, Lille, France). For a substantial subset of the analyzed samples, sequence chromatograms displayed overlapping peaks, suggesting mixed infections/colonizations with at least two distinct *Blastocystis* sp. STs, which could not be individually resolved using Sanger sequencing. SSU rDNA gene sequences from samples with single *Blastocystis* sp. infections/colonizations were deposited in GenBank under accession numbers PV866640–PV866661. These sequences were compared to reference sequences for all known *Blastocystis* sp. STs available in the National Center for Biotechnology Information (NCBI) database using the Nucleotide Basic Local Alignment Search (BLASTN) tool (https://blast.ncbi.nlm.nih.gov/Blast.cgi). The ST of these isolates was determined based on an exact or closest match with the reference sequences. Sequences from isolates belonging to the same ST were then aligned with each other using BioEdit v7.7.1 (https://bioedit.software.informer.com/) to assess intra-ST diversity and to determine the so-called genotypes or genetically distinct strains within the same ST for inclusion in the subsequent phylogenetic analysis as previously performed (Guilavogui et al., 2022; Naguib et al., 2024).

### 2.4 Phylogenetic analysis of *Blastocystis* sp isolates

To validate the STs determined by BLASTN for all isolates identified in this survey from single infections/colonizations in oyster and environmental samples using phylogenetic inference, the full-length SSU rDNA gene reference sequences of the 45 *Blastocystis* sp. STs validated to date were initially extracted from the GenBank database (Figueiredo et al., 2025) then aligned using MAFFT v7.490 (https://mafft.cbrc.jp/alignment/software/) with the iterative refinement L-INS-i method (Katoh and Standley, 2013). Briefly, the maximum likelihood (ML) phylogenetic tree was constructed from this untrimmed alignment using the IQ-TREE software (http://www.iqtree.org/), with the substitution model K2P + I + G4 and unequal transition/transversion rate and equal base frequency (Nguyen et al., 2015). No trimming was applied to the reference alignment, as exploratory analyses showed that the topology obtained from the full-length reference sequences remained essentially unchanged with or without trimming. Branch reliability of the ML tree was assessed with 1000 bootstrap replicates. This tree was rooted on the cluster composed of ST15 and ST28, identified as the most basal lineage within the *Blastocystis* genus in recent phylogenetic investigations (Koelher et al., 2024; Santin et al., 2024; Figueiredo et al., 2025). In a second step, the nine partial SSU rDNA gene sequences representative of all the genotypes identified in the present study by Sanger sequencing were added to the same MAFFT reference alignment and subsequently placed within the fixed reference ML tree using the highly scalable tool EPA-ng (Barbera et al., 2019), which computes the most likely placement of each query sequence. The resulting placements were then analyzed and visualized with Gappa (Czech et al., 2020). Additional visual enhancements and annotations of the tree were performed using the Interactive Tree Of Life (iTOL) v7 platform (https://itol.embl.de/) (Letunic and Bork, 2021).

### 2.5 Subtyping of *Blastocystis* sp. isolates from mixed infections/colonizations using next-generation amplicon sequencing

Amplicons targeting the SSU rDNA gene region, derived from selected oyster and environmental samples identified as harboring mixed *Blastocystis* sp. infections/colonizations via Sanger sequencing, were subjected to high-throughput sequencing using a MinION device (Oxford Nanopore Technologies, Oxford, UK). Briefly, sequencing libraries were prepared with the Native Barcoding Kit (Oxford Nanopore Technologies) following the manufacturer's instructions. Amplicons were end-repaired and dA-tailed using the NEBNext Ultra II End Repair/dA-Tailing Module (NEB, Ipswich, MA, USA), barcoded with the Blunt/TA Ligase Master Mix (NEB), and ligated to sequencing adapters using the NEBNext Quick Ligation Module (NEB). After purification, libraries were loaded onto a MinION flow cell and sequenced on the nanopore platform. After removal of barcodes and sequencing adapters, reads were initially screened and taxonomically classified as *Blastocystis* sp. using BLASTN against the 18S rDNA SILVA database (version 138; https://www.arb-silva.de/). Reads identified as *Blastocystis* sp. were then assigned to specific STs via a second BLASTN search against a custom reference database containing homologous sequences from all 45 currently known STs, using a stringent *E*-value cutoff of 1e-100. STs representing less than 1% of the total reads in a given sample were excluded from the analysis. The sequences generated by next-generation sequencing (NGS) were submitted to NCBI Sequence Read Archive (SRA) under the Bioproject PRJNA1357851.

## 3 Results and discussion

### 3.1 qPCR detection and prevalence of *Blastocystis* sp*.* in oysters and environmental samples

The present study included a retrospective analysis of oysters collected in 2021 from various French production basins, followed by a targeted survey conducted in 2024 at the Marennes-Oléron site. In parallel, seawater samples collected near oyster harvesting areas in 2021 were also analyzed to assess the presence of the parasite in the marine environment. Regarding oysters, the prevalence of *Blastocystis* sp. detected by qPCR assay in the batch collected in 2021 across all three production sites was very low, at only 1.4% (5/360), with four positive samples from Arcachon Bay in March and July (4/120; 3.3%), one from Marennes-Oléron Bay in July (1/120; 0.8%) and none from Bourgneuf Bay (0/120) (Table 1). For the sampling of 120 oysters from 2024 (Table 2), 12.5% (15/120) were positive in the gills, 10% (12/120) in the digestive tract, 3.3% (4/120) in both organs, and 19.2% (23/120) in one or both of these organs. None of the oysters collected in September 2024 tested positive. The substantial difference in the overall prevalence of *Blastocystis* sp. between the two oyster sampling campaigns (1.4% in 2021 vs. 19.2% in 2024; Fisher's exact test, *P* < 0.05) is highly significant, and becomes even more striking when focusing solely on the shared sampling site of Marennes-Oléron Bay (0.8% in 2021 vs. 19.2% in 2024; Fisher's exact test, *P* < 0.05). A first factor that could explain the retrospectively low prevalence observed in 2021 is potential DNA degradation, likely resulting from long-term storage that may have altered the quantity/quality of protozoan DNA and compromise the performance of the qPCR. Nonetheless, other contributing factors cannot be ruled out, such as differences in the type of tissues analyzed (pooled gills, digestive gland, and mantle in 2021 vs. individual gills and digestive tract in 2024), or in the DNA extraction protocols. Indeed, the extraction method used in 2024 was notably optimized to enhance parasite detection, incorporating a mechanical lysis step with ceramic beads to improve the disruption of cystic forms of *Blastocystis* sp., as well as a more effective removal of PCR inhibitors. It is also plausible that the COVID-19 pandemic (2020−2021) likely led to a decline in the circulation of various waterborne infectious pathogens potentially contaminating mollusks, including some zoonotic agents, as a result of widespread public health interventions (Liu et al., 2024).

**Table 1:** Oyster and environmental samples collected in 2021 positive for *Blastocystis* sp. by qPCR and subtyping of the corresponding isolates by Sanger sequencing and NGS.

Sample No., place and date of sampling	qPCR assay	Subtype and genotype (Sanger sequencing)	Subtype (NGS)
Oysters			
2114522 Marennes-Oléron 07/21	+	ST44-1	
2105109 Arcachon 03/21	+	ST4-1	
2105110 Arcachon 03/21	+	ST26-1	
2105111 Arcachon 03/21	+	ST4-1	
2114223 Arcachon 07/21	+	ST4-1	
Nanoplankton			
P7D2 Bourgneuf 07/21	+	MI^a^	
P7G9 Marennes-Oléron 11/21	+	MI^a^	ST4 + ST6 +ST7 + ST28
Meso- and microplankton			
P8A3 Arcachon 03/21	+	ST7-2	
P8E6 Bourgneuf 11/21	+	MI^a^	

a MI, Mixed infection/colonization with unidentified STs by Sanger sequencing.

**Table 2:** Frequency of *Blastocystis* sp. detected in different oyster organs collected in 2024 from the Marennes-Oléron Bay.

Sample collection date	Gills	Gastrointestinal tract	Both organs	Infected oysters (gills and/or gastrointestinal tract)
07/2024	9/40 (22.5%)	8/40 (20.0%)	4/40 (10.0%)	13/40 (32.5%)
09/2024	0/40 (0%)	0/40 (0%)	0/40 (0%)	0/40 (0%)
11/2024	6/40 (15.0%)	4/40 (10.0%)	0/40 (0%)	10/40 (25.0%)
Total	15/120 (12.5%)	12/120 (10.0%)	4/120 (3.3%)	23/120 (19.2%)

It is noteworthy that the 19.2% prevalence observed in oysters from Marennes-Oléron Bay in 2024 is substantially lower than the frequencies reported in Mexico: 71.3% and 77% in *Crassotrea virginica* oysters obtained from markets in Mexico City (Martinez Barbabosa et al., 2017, Martinez Barbabosa et al., 2021), and 67% in samples from the Actopan River, Veracruz (Campos Compean et al., 2018). However, these high prevalence rates reported in the Mexico area should be interpreted with caution, as parasite identification was based on microscopic observation of the intestinal contents of the oysters. Indeed, microscopic identification is particularly challenging considering the small size of the cystic form of the parasite, which can easily be misidentified as fecal debris, potentially leading to an overestimation or underestimation of its true prevalence. Moreover, the vegetative stages of *Blastocystis* sp. can rapidly degrade and become unrecognizable under the microscope. Therefore, minimizing the time of fixation is essential to preserve high diagnostic sensitivity when using microscopy (Bart et al., 2013). Consequently, and despite the potential high number of microscopically positive samples in Mexico (around 575 from three studies), only four were confirmed as *Blastocystis* sp.-positive by end-point PCR followed by Sanger sequencing (Campos Compean et al., 2018; Martinez Barbabosa et al., 2018, Martinez Barbabosa et al., 2021).

Consistent with observations from oysters collected in 2021, the prevalence of *Blastocystis* sp. in environmental samples from the same year remained very low, likely reflecting the factors discussed above. Only two nanoplankton small-size fractions (1–20 μm), potentially containing the free cystic forms (generally 2–5 μm) of *Blastocystis* sp., from the Marennes-Oléron and Bourgneuf bays tested positive for the parasite (2/130; 1.5%). In addition, two large-size fractions (20–200 μm) of crushed micro-mesoplanktonic organisms from the Bourgneuf and Arcachon bays tested positive (2/80; 2.5%). Overall, *Blastocystis* sp. was detected in only four of the 210 environmental samples analyzed (1.9%) (Table 1). However, this protozoan has already been reported at potentially significant frequencies in various water sources, mainly rivers or seawater, but also in drinking water, tanks, ponds, and wastewater as recently reviewed (Hublin et al., 2021; Attah et al., 2023; Mahdavi et al., 2025). Additionally, *Blastocystis* sp. was detected in subsurface seawater samples from the eastern English Channel, collected at an offshore marine station located 8 km from the coast (Ryckman et al., 2024), further highlighting the widespread presence of this parasite in diverse water sources even far from the shoreline. To the best of our knowledge, this study is also the first to report the presence of *Blastocystis* sp. in marine micro-mesoplankton (Ghafari-Cherati et al., 2024), a community primarily composed of crustaceans (mainly copepods and cladocerans) and large protozoans such as ciliates and dinoflagellates. This finding highlights the need for further research to identify additional potential hosts or carriers of the parasite within this planktonic compartment, which may thus act as vectors/reservoirs within the food web and facilitate transmission to edible marine fish and marine mammals already known to harbor *Blastocystis* sp. (Gantois et al., 2020; Asghari et al., 2024).

As part of our study, it was not possible to generate qPCR quantification curves to determine the *Blastocystis* sp. load in the positive oysters and environmental samples. However, it could still be roughly estimated based on the mean Ct values obtained herein and by extrapolating them, using the quantification curves generated by qPCR assays also targeting the SSU rDNA gene from dilution series of cultured ST3 (Šloufová et al., 2022; Šejnohová et al., 2024). For the oyster samples, Ct values from the 2021 sampling campaign ranged from 21 to 27, and for the 2024 campaign, from 21 to 35, with more than half of the values above 30. In plankton samples, Ct values ranged from 23 to 34. According to the quantification curves established for ST3, Ct values between 21 and 28 correspond to a moderate protozoan load, whereas values above 29 indicate a low load. No high protozoan load (Ct < 20) was detected in our study, suggesting a low intensity of parasites presence, particularly in oysters. This pattern is consistent with colonization rather than a true infection, likely resulting from environmental fecal contamination coupled with the high water filtration capacity of these mollusks, as previously proposed for mussels (Ryckman et al., 2024).

### 3.2 ST distribution of *Blastocystis* sp. in oysters and environmental samples based on Sanger sequencing data

The second step of this study was to purify and sequence, using the Sanger method, the qPCR products from the 36 oysters and environmental samples that tested positive for *Blastocystis* sp., in order to identify the STs present (Table 1, Table 3). Among all samples of diverse origin, analysis of the sequence chromatograms revealed that 14 (38.9%) showed mixed infections/colonizations meaning the presence of at least two different STs within the same sample. Specifically, for oysters collected in 2024 from the Marennes-Oléron site, the frequency of mixed infections/colonizations was notably high, reaching approximately 40% (11 out of 26). This is comparable to the rate observed in mussels from northern France in 2023 (around 50%) (Ryckman et al., 2024). More broadly, such high frequencies of mixed infections/colonizations have also been reported across numerous animal groups (Hublin et al., 2021) and are likely due to multiple contamination sources of varied origin within a geographically confined environment.

**Table 3:** Oysters collected in 2024 positive for *Blastocystis* sp. by qPCR in the gills and/or gastrointestinal tract and subtyping of the corresponding isolates by Sanger sequencing and NGS. The oyster samples with code 1-H were collected in July, while those with codes 3-H were collected in November.

Sample No Gills and sampling date	qPCR assay	Subtype and genotype (Sanger sequencing)	Sample No. Gastrointestinal tract and sampling date	qPCR assay	Subtype and genotype (Sanger sequencing)	Subtype (NGS)
1-H13B 07/24	+	ST3–3	1-H13T 07/24	−		
1-H16B 07/24	−		1-H16T 07/24	+	MI^a^	ST1 + ST3 + ST4 + ST6 + ST7
1-H19B 07/24	−		1-H19T 07/24	+	ST3–1	
1-H25B 07/24	+	MI^a^	1-H25T 07/24	+	ST7–1	
1-H27B 07/24	−		1-H27T 07/24	+	MI^a^	ST3 + ST4 + ST7 + ST21 + ST26 + ST30
1-H28B 07/24	+	ST7–2	1-H28T 07/24	−		
1-H29B 07/24	+	MI^a^	1-H29T 07/24	+	MI^a^	
1-H30B 07/24	−		1-H30T 07/24	+	ST3–2	
1-H31B 07/24	+	MI^a^	1-H31T 07/24	+	MI^a^	
1-H35B 07/24	+	ST4–1	1-H35T 07/24	+	MI^a^	
1-H36B 07/24	+	MI^a^	1-H36T 07/24	−		
1-H37B 07/24	+	ST3–4	1-H37T 07/24	−		
1-H38B 07/24	+	MI^a^	1-H38T 07/24	−		
3-H8B 11/24	+	ST7–1	3-H8T 11/24	−		
3-H10B 11/24	−		3-H10T 11/24	+	MI^a^	ST1 + ST2 + ST4 + ST6 + ST7
3-H11B 11/24	+	ST7–1	3-H11T 11/24	−		
3-H13B 11/24	+	ST7–1	3-H13T 11/24	−		
3-H16B 11/24	+	ST7–1	3-H16T 11/24	−		
3-H22B 11/24	+	ST7–1	3-H22T 11/24	−		
3-H24B 11/24	+	ST7–1	3-H24T 11/24	−		
3-H35B 11/24	−		3-H35T 11/24	+	ST7–1	
3-H38B 11/24	−		3-H38T 11/24	+	ST7–1	
3-H40B 11/24	−		3-H40T 11/24	+	ST7–1	

a MI, Mixed infection/colonization with unidentified STs by Sanger sequencing.

**Fig. 2:**
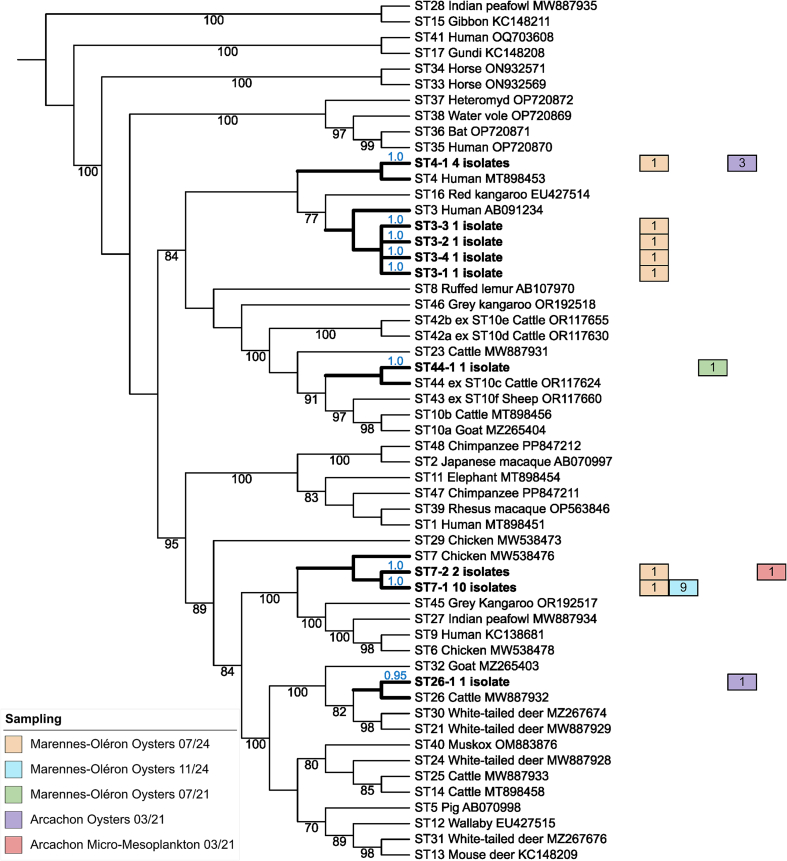
Maximum-likelihood phylogenetic tree of *Blastocystis* sp. isolates based on partial SSU rDNA gene sequences. The tree is rooted with reference sequences from the earliest diverging cluster (ST15/ST28) within the *Blastocystis* genus. Sequences generated in this study by Sanger sequencing from oysters and environmental samples are shown in bold. Colored boxes indicate the number of isolates for each genotype, as well as their origin and geographical source, as described in the legend. Accession numbers of reference sequences representing known STs are provided. Bootstrap support values (in black) are shown at the nodes; values below 70% are omitted. Values in blue next to each analyzed isolate represent the likelihood of that sequence being placed at the corresponding node. (For interpretation of the references to colour in this figure legend, the reader is referred to the web version of this article.)

The sequence profiles of the remaining 22 oysters and plankton samples indicated single infections/colonizations by Sanger technology and the subtyping of the corresponding isolates was thus initially performed by querying the NCBI database. The obtained sequences shared 99.3% to 100% identity with homologous reference sequences of known STs, allowing their ST identification (Table 1, Table 3). In parallel, due to the known potential ST misclassification of certain isolates in the databases (Stensvold and Clark, 2020), these sequences were also included in a phylogenetic analysis to confirm the results obtained by BLASTN, according to likelihood values of clustering (0.95 to 1.0) (Fig. 2). Regarding the oysters, three isolates of 2021 from Arcachon and one of 2024 from Marennes-Oléron belonged to ST4 and showed 100% sequence identity, leading to the characterization of a single genotype (ST4-1). One isolate was also identified as ST26 from an oyster harvested in 2021 at the Arcachon bay (genotype ST26-1), and another as ST44 (genotype ST44-1), found in an oyster of the Marennes-Oléron sampling site collected in 2021. Among the remaining isolates, four were reported in oysters of 2024 from Marennes-Oléron bay as belonging to ST3. The corresponding sequences exhibited 98.6% to 99.3% identity among them and were all different, leading to the identification of four genotypes (ST3-1 to ST3-4). Finally, the last identified ST was ST7, which was predominant in our survey with a total of 11 isolates found in oysters from Marennes-Oléron collected in 2024, as well as in one micro-mesoplankton sample from Arcachon Basin sampled in 2021. Based on sequence comparisons, two genotypes were identified (ST7-1 and ST7-2), which, strikingly, shared only 94.4% sequence identity. However, a 4% sequence divergence cut-off has been proposed between STs (Stensvold and Clark, 2020), suggesting that ST7 likely should be divided into at least two different STs based on partial sequences of the SSU rDNA gene. To confirm the validity of this ST division, near-complete SSU rDNA gene sequences of the isolates of interest need to be further generated and then compared to homologous full-length reference sequences identified as ST7.

### 3.3 *Blastocystis* sp. ST identification using next-generation sequencing

To gain a deeper understanding of the diversity of *Blastocystis* sp. STs in the Marennes-Oléron Bay, four qPCR-positive samples were analyzed using a NGS approach. These included PCR products from three oyster gastrointestinal tracts collected in 2024 (1-H16T, 1-H27T, and 3-H10T) and one nanoplankton sample collected in 2021 (P7G9), all of which showed evidence of mixed infections/colonizations based on Sanger sequencing. NGS analysis of the oyster samples revealed a total of nine distinct STs: ST1, ST2, ST3, ST4, ST6, ST7, ST21, ST26, and ST30 (Table 3). The relative abundance of reads varied considerably among samples and STs. ST7 was overwhelmingly dominant in two oyster samples (1-H16T and 3-H10T), representing 84% and 86% of reads, respectively. In contrast, ST4 and ST30 were the most abundant STs in the third oyster sample (1-H27T), each accounting for approximately 35% of reads. Notably, ST4 and ST7 were the only STs detected in all three oyster samples. In the nanoplankton sample, four STs were identified—ST4, ST6, ST7, and ST28 (Table 1)—with ST4 and ST7 being predominant, comprising 47% and 48% of reads, respectively.

All cumulative data from Sanger and NGS subtyping provide a comprehensive overview of the sources and circulation of *Blastocystis* sp. isolates present both in oysters and in their surrounding marine environment. In the Marennes-Oléron Bay alone—the main focus of our study—11 STs were identified, either in oysters collected in 2024 (ST1, ST2, ST3, ST4, ST6, ST7, ST21, ST26, ST30), in 2021 (ST44), or within a nanoplankton fraction (ST4, ST6, ST7, ST28). The diversity of STs identified, particularly in oysters—as well as that recently reported in mussels (Ryckman et al., 2024)—does not highlight any predominant ST among mollusks, thus strongly suggesting that these animals are not natural hosts of *Blastocystis* sp. Moreover, due to their exceptional filtration capacity, feeding bivalves efficiently concentrate particulate matter from the surrounding seawater, including fecal contaminants and protozoa, thereby functioning as carriers of these microorganisms. Furthermore, in the Marennes-Oléron Bay, three STs (ST4, ST6, and ST7) are shared between oysters and their environment (nanoplankton), making these mollusks true sentinels of the microbiological quality and diversity of their ecosystem.

### 3.4 Hypotheses on the origins of pollution sources impacting oysters and their marine environment

The STs identified in this study provide a basis for formulating plausible hypotheses concerning the origins of the multiple sources of pollution affecting oysters and their surrounding environment. Indeed, although ST1 to ST3 have been reported in various animal groups at variable frequencies (Hublin et al., 2021), they remain predominantly associated with humans, particularly ST3, which is the most frequently detected ST worldwide (Alfellani et al., 2013a). Furthermore, ST1 was the only ST identified in oysters from Mexico (Campos Compean et al., 2018; Martinez Barbabosa et al., 2021). ST4 is another ST primarily colonizing humans, but it displays a distinct geographically restricted distribution (Alfellani et al., 2013a). In fact, ST4 is especially common in Europe, where it can represent up to 20% of human isolates (El Safadi et al., 2016; Muadica et al., 2020), suggesting a recent emergence on the European continent (Clark et al., 2013). Interestingly, the ST4–1 genotype identified in the present study in oysters from both Marennes-Oléron and Arcachon bays exhibited 100% sequence identity with human isolates collected in several French hospitals (El Safadi et al., 2016; Menu et al., 2019). The same genotype has also been reported in a seal stranded on the northern coast of France, suggesting a wide dissemination across hosts and environments (Gantois et al., 2020). It is therefore highly probable that the presence of these first four STs (ST1 to ST4) in oysters results from seawater contamination by human fecal matter containing *Blastocystis* sp. cysts. As shown on the map of Marennes-Oléron Bay (Fig. 1), a sewage treatment plant located near the oyster harvesting area could represent a potential source of such contamination, since its effluents are indirectly discharged into the bay. The detection of ST4 in the seawater nanoplankton fraction sampled near the oysters in the same area further supports this hypothesis.

Regarding the other STs identified in our study, two of them, ST6 and ST7, are recognized as avian STs, due to their strong predominance in birds (Greige et al., 2018; Rauff-Adetotun et al., 2021; Hublin et al., 2021). Notably, ST7 was the most prevalent ST among *Blastocystis* sp. isolates obtained from oysters in Marennes-Oléron in 2024, and it was also detected in a micro-mesoplankton fraction collected in Arcachon in 2021. Moreover, co-colonization involving ST6 and ST7 was detected by NGS both in the Marennes-Oléron oyster samples of 2024 and in the nanoplankton fraction of 2021 collected from the same bay. These findings strongly suggest that the occurrence of these avian STs in oysters and in the surrounding aquatic environment is likely linked to seawater contamination by bird droppings. Interestingly, most ST7 isolates identified in both oysters and the nanoplankton fraction from Marennes-Oléron originated from samples collected in March and November, periods corresponding to the wintering season of migratory birds such as the Dark-bellied Brent goose (*Branta bernicla bernicla*), which forms large colonies along the French Atlantic coast, particularly around Île d'Oléron. Feeding mainly on marine vegetation (eelgrass, sea lettuce, and mosses) at low tide, these geese were observed daily near our sampling sites in Marennes-Oléron, where they likely released fecal matter directly into the water. Future investigations on the feces of these birds could help confirm their role as a potential reservoir contributing to environmental contamination by *Blastocystis* sp.

Other STs have also been reported in the Marennes-Oléron bay, namely ST21, ST26, ST30, and ST44 in oysters, and ST28 in a nanoplankton fraction. The same ST26 isolate was also found in an oyster from the Arcachon basin. These five STs have mainly been identified in cattle and small ruminants worldwide and are considered adapted to these animal groups (Maloney et al., 2019; Santin et al., 2023; Figueiredo et al., 2024; Naguib et al., 2024; Santin et al., 2024; Figueiredo et al., 2025). In addition to being an active area for seafood production, Marennes-Oléron and its surroundings are also agricultural land, particularly with cattle farms. Consequently, it is highly probable that these *Blastocystis* sp. isolates originated from ruminants and that their feces may have been carried by agricultural run-off from farms into streams flowing into the Marennes-Oléron Bay, leading to the contamination of the oysters. A similar scenario has been proposed for mussels contaminated by ST26 and ST44 on the northern coast of France (Ryckman et al., 2024).

### 3.5 Estimation of the risk of transmission to humans through the consumption of oysters infected with *Blastocystis* sp.

Consumed raw, oysters contaminated with *Blastocystis* sp. represent a potential risk to consumers. However, to accurately estimate this risk, it is first critical to determine whether the ten STs identified in these mollusks (ST1, ST2, ST3, ST4, ST6, ST7, ST21, ST26, ST30, and ST44) are known to be transmissible to humans. This question does not arise for ST1 to ST4, which are highly predominant in the human population (Alfellani et al., 2013a). The same applies to the avian ST6 and ST7, which account for about 10% of human isolates (Stensvold and Clark, 2016) and whose zoonotic potential has been clearly demonstrated (Greige et al., 2018). Regarding ruminant STs, ST26 has already been reported in humans in epidemiological surveys conducted in Vietnam (Nguyen et al., 2023), Egypt (Naguib et al., 2023), and Senegal (Khaled et al., 2020), as well as ST44, which has been identified in Vietnam (Nguyen et al., 2023), Egypt (Naguib et al., 2023), and in Syrian refugee camps in Lebanon (Khaled et al., 2021). Conversely, to our knowledge, no studies have yet identified ST21 and ST30 in the human population. Overall, the risk to the population is high since eight of the ten STs found in these mollusks are potentially transmissible to humans, making oysters an important reservoir of contamination through their consumption.

This risk assessment must also consider the viability and multiplication of *Blastocystis* sp. within its bivalve host. Unfortunately, no data are currently available regarding its survival or replication in mollusks. However, cystic forms of the parasite are known to persist for several weeks in water at room temperature and to resist common water disinfection treatments such as chlorination and ozonation (Moe et al., 1996; Tan, 2008). Further histological analyses of gastrointestinal tissues from positive animals should also be conducted to determine whether the parasite can multiply within bivalve hosts. The previously observed Ct values, together with the distribution of the identified STs, instead suggest that mollusks primarily carry cystic forms of the parasite, reflecting fecal contamination of human or animal origin in the surrounding environment.

## 4 Conclusions

This survey constitutes the most comprehensive molecular study to date on the distribution of *Blastocystis* sp. STs in oysters, improving understanding of the parasite's epidemiology in bivalves and its circulation in marine environments. The ST distribution determined herein in wild oysters and more broadly among mollusks (Ghafari-Cherati et al., 2024; Ryckman et al., 2024), suggests that these marine animals are likely passive carriers of *Blastocystis* sp. isolates originating from human or animal waste rather than natural hosts, highlighting their potential as sensitive indicators of fecal contamination in aquatic environments. Several hypotheses regarding the origin of this contamination in oysters and their seawater environment need to be confirmed through a One Health approach combining the screening of human, animal (cattle and birds), and environmental samples (e.g., wastewater treatment effluents, streams near farms, among others) within a restricted geographic area such as the Marennes-Oléron Bay. The STs identified, being largely transmissible to humans, indicate that the risk of contamination for consumers of raw shellfish and particularly oysters is real, even though the viability and possible infectivity of the parasite in this host still need to be clarified. Human and animal fecal pollution most likely remains the main source of contamination in this marine environment, especially along coastlines and in oyster production bays, where uncontrolled waste discharges contribute to the presence of intestinal protozoa such as *Blastocystis* sp.

Exposure “from sea to fork” can be mitigated through the regulatory depuration process commonly used in the shellfish industry, during which farmed mollusks are immersed in clean seawater for a variable period before sale and consumption, allowing them to naturally eliminate contaminants, assuming that the seawater used is itself free of protozoa. However, the effectiveness of this process has proven somewhat inconsistent, for example with *Cryptosporidium*, and appears to depend on specific factors related to the depuration procedure or the species of shellfish to be decontaminated (Willis et al., 2014). This process should also be tested in order to evaluate its efficiency against *Blastocystis* sp. in farmed oysters. No cases of *Blastocystis* sp. infection linked to oyster consumption have yet been documented in the literature, which can easily be explained by the lack of systematic investigation for pathogenic protozoa in the stool samples of patients with transient digestive disorders. This suggests that such contamination cases may be largely underreported, especially when considering the significant prevalence of this parasite in these mollusks. Therefore, further research should be undertaken to improve our understanding of the contamination dynamics of seafood by protozoa potentially pathogenic to humans. This is particularly important given that current climate change through the combination of factors such as rising temperatures, more frequent rainfall, and flooding, may compromise water quality and increase the risk of outbreaks of waterborne protozoa such as *Blastocystis* sp., which can affect filter-feeding bivalves.

## CRediT authorship contribution statement

**Manon Ryckman:** Writing – review & editing, Methodology, Investigation, Formal analysis, Data curation. **Constance Denoyelle:** Writing – review & editing, Methodology, Investigation, Formal analysis, Data curation. **Cyrielle Lecadet:** Writing – review & editing, Methodology, Investigation, Formal analysis, Data curation. **Nausicaa Gantois:** Methodology, Investigation, Formal analysis, Data curation. **Jeremy Desramaut:** Methodology, Investigation, Formal analysis, Data curation. **Ruben Garcia Dominguez:** Writing – review & editing, Software, Resources, Methodology, Data curation. **Magali Chabé:** Methodology, Investigation, Formal analysis, Data curation. **Luen-Luen Li:** Writing – review & editing, Software, Resources, Methodology, Investigation, Data curation. **Sébastien Monchy:** Writing – review & editing, Validation, Supervision, Project administration, Investigation, Funding acquisition, Conceptualization. **Gabriela Certad:** Writing – review & editing, Validation, Supervision, Project administration, Funding acquisition, Conceptualization. **Isabelle Arzul:** Writing – review & editing, Validation, Supervision, Project administration, Funding acquisition, Conceptualization. **Eric Viscogliosi:** Writing – review & editing, Writing – original draft, Validation, Supervision, Project administration, Funding acquisition, Conceptualization.

## Funding sources

This study was supported by the 10.13039/501100004794Centre National de la Recherche Scientifique (CNRS), the 10.13039/501100001677Institut National de la Santé et de la Recherche Médicale (Inserm), the 10.13039/501100003762Institut Pasteur of Lille, the University of Lille, the 10.13039/100009111Institut Français de Recherche pour l'Exploitation de la Mer (Ifremer), the University of Littoral Côte d'Opale and the CHRU of Lille, France. This work is also supported by the graduate school IFSEA that benefits from a France 2030 Grant (ANR-21-EXES-0011) operated by the French National Research Agency and by the French State and the French Region Hauts-de-France in the framework of the project CPER IDEAL 2021–2027. M.R. was supported by a PhD fellowship from the Region Hauts-de-France and the Pôle Métropolitain de la Côte d'Opale (PMCO) and C.D. from the University of Lille.

## Declaration of competing interest

The authors declare that they have no known competing financial interests or personal relationships that could have appeared to influence the work reported in this paper.

## Data Availability

All relevant data are within the paper.
